# Comparative blood transcriptome analysis reveals changes in immunity, and transcripts related to metabolism and development in the critically endangered Yangtze finless porpoise (*Neophocaena asiaeorientalis asiaeorientalis*) with age

**DOI:** 10.1016/j.cirep.2025.200255

**Published:** 2025-10-03

**Authors:** Syed Ata Ur Rahman Shah, Haobo Zhang, Bin Tang, Dekui He, Ghulam Nabi, Jinsong Zheng, Yujiang Hao

**Affiliations:** a Institute of Hydrobiology, Chinese Academy of Sciences, Wuhan 430072, China; b University of Chinese Academy of Sciences, Beijing 100049, China; c National Aquatic Biological Resource Center, Wuhan 430072, China; d Center for Animal Sciences and Fisheries, University of Swat, Khyber Pakhtunkhwa, Swat, 19200, Pakistan

**Keywords:** Yangtze finless porpoise, Blood, Immune system, Aging, Disease

## Abstract

The Yangtze finless porpoise (YFP), a critically endangered small odontocete species, is mostly living in the lower and middle sections of the Yangtze River and its two big adjacent lakes. The YFP population experienced a drastic decline due to the threats from various human activities. Comprehending their biology, particularly the immune changes associated with aging, is essential for ensuring their protection. The study aimed to identify genes and pathways in thirteen YFPs blood that are influenced by age, revealing their immune system’s susceptibility to aging and reduced disease response. The current research utilized RNA-Seq to find differentially expressed genes (DEGs) in blood tissues, and we screened 478, 442, and 739 DEGs in comparison groups of calf vs. adult, calf vs. old, and adult vs. old, respectively. STEM, GO and KEGG enrichment analyses revealed significant changes in metabolism, immune-related, development, signal transduction pathways, and aging among the three age-related groups. In the calf vs. adult group, the DEGs were mainly associated with primary immunodeficiency, IgA production, and B cell receptor signaling pathways. In the calf vs. old group, the DEGs were significantly enriched in autoimmune disease, cytokine interactions, and viral/bacterial infections. In the adult vs. old group, the DEGs were primarily linked to cytokine interactions and bile secretion. The current study identifies DEGs affecting immunity, development, and growth in YFPs by comparing blood transcriptomes of calves, adults, and old YFPs. This information provides a basis for studying YFP’s development, growth, and aging and will guide future research on disease prevention, treatment strategies, management, and conservation of YFP.

## Highlights

• The present study utilized RNA-Seq to find differentially expressed genes in the blood tissues of thirteen critically endangered Yangtze finless porpoises with aging.
• A total of 478, 442, and 739 differentially expressed genes were identified in calf vs. adult, calf vs. old, and adult vs. old comparisons, respectively.
• STEM analysis reveals significant alterations in immune, development, metabolism, and signal transduction pathways in the different age groups.
• The current study provides a basis for studying Yangtze finless porpoises development, growth, and aging.

## Introduction

The Yangtze finless porpoise, a small cetacean exclusively found in the lower and middle regions of the Yangtze River and its two big adjacent lakes (the Poyang Lake and Dongting Lake), and is now facing a population decline due to ecological degeneration of the Yangtze River, despite a 23.4 % increase in 2022 [40,48,73]. The species is essential for evaluating the richness and overall health of the freshwater Yangtze River environment [75]. Dams, sand mining, heavy shipping, and engineering development projects involving water pose a threat to the YFP population. Due to its rapid decline and need for urgent research and protection, the YFP was classified as “Critically Endangered” by the International Union for the Conservation of Nature’s Species Survival Commission since 2013 [63], it was also upgraded as a national first-class protected wild animal in 2021 under the *List of National Key Protected Wild Animals*.

Transcriptome sequencing enables real-time gene expression detection, identifying metabolic pathways and genes related to particular traits [61]. However, few studies have identified molecular markers in YFP blood using transcriptome, including those related to age and different habitats, due to difficulties in obtaining fresh tissues from rare and protected species [4,16,[39], [40], [41],^69^]). Blood expresses 80 % of the genes present in essential tissues including the heart, brain, and liver, making it essential for animal physiological functions and life cycles and facilitating the development of molecular markers [24,38,55]. Age-related gene variations in blood have been observed in Japanese black cattle, humans, pandas, and African green monkeys [3,5,14,34]. Aging leads to senescence, a process involving changes in metabolism and immune system. Gene expression levels vary with age, making aging a significant risk factor for infectious diseases, though the molecular mechanisms behind this process remain largely unknown [49,50,53]. Immunosenescence, characterized by immune changes with age, is observed in humans, mice, zebra finches, and wolves [6,20,47,51,53]. Overexpression of immune response and inflammatory genes and under expression of energy metabolism genes were found in a meta-analysis of age-related gene expression patterns [11]. Aging Tibetan macaques, wolves, and giant pandas exhibit gene expression changes that affect immune responses, inflammation, and metabolism [6,14,67]. These findings suggest that improving mitochondrial function or restricting glycolysis can prolong lifespan and support healthy aging in a variety of species [17]. Although there is little information on how YFPʹs immunological and metabolic functions vary with age, recent transcriptome studies have identified DEGs linked to immunity in the blood [13,14,69].

The current study aims to better understand immune-related gene modifications and examine gene and pathway changes at different YFP growth and development ages. Using RNA-seq technology, age related DEGs were identified in the YFP blood samples, and their functional enrichment was determined. The study explored the age-related changes in YFPʹs immune system, development, and metabolism, highlighting the importance of YFP research for future *ex-situ* protection and artificial breeding.

## Materials and methods

### Ethical statement

The present research adhered to China’s national regulations and institutional policies for animal care and use, with medical examination and experiments approved by the Institute of Hydrobiology (IHB/LL/2022-YFF1301604), Chinese Academy of Sciences, Wuhan, and in compliance with the Chinese Aquatic Animal Protection Act.

### Animals and sample collection

In the present research, 13 YFP blood samples were taken from the largest *ex-situ* YFP population in Tian-e-Zhou Oxbow, Hubei Province, China; see Table S1 for specific information. Thirteen blood samples, using a body length and age estimate method [18], the animals were categorized into three groups on the basis of a previous study [69]: six from the calf group (1–6 years old), four from the adult group (7–12 years old), and three from the old group (≥13 years old). In the current study, we used conventional “sound chase and net capture” strategy to catch the YFPs in batches. We used soft nets to build an enclosure and turned off the motorboat to relax the animals [22]. A follow-up physical examination was conducted once the animals were calm and moving freely with no obvious signs of stress. With the use of a disposable syringe (10 mL), blood samples were drawn from the main tail vein and transferred to a vacuum collection vessel (PAXgeneTM, BD, USA), and then they were added into cryopreservation tubes, added with Trizol at a 1:3 (blood: Trizol = 1:3) ratio, and then preserved in liquid nitrogen.

### The extraction of tRNA, mRNA sequencing, and analysis

Total RNA was extracted from thirteen YFPs’ entire blood samples using the PAXgene Blood RNA Kit (QIAGEN, Germany). We measured the RNA quality using an Agilent 2100 Bioanalyzer (Agilent Santa Clara, CA, USA). We prepared the cDNA library using the MGIEasy RNA library preparation kit. High-throughput RNA sequencing was performed on the MGI-2000 platform using 2 × 150 bp paired-end reads following library preparation. Using SOAPnuke software (v1.4.0), the sequencing data was filtered to exclude reads containing sequencing adapters [36]. The YFP reference genome (https://www.ncbi.nlm.nih.gov/genome/?term=Neophocaena %20asiaorientalis) was used for sequencing the clean reads after they had been processed using HISAT2 software (v1.4.0) [32]. Using RSEM software (v1.2.8), gene expression levels were determined and reported as FPKM, or fragments per kilobase of transcript per million mapped fragments [35].

### Power analysis calculation and biological replicates

The statistical power of the experimental design, computed using RNASeqPower, was 0.92. To ensure adequate detection of differential expression, three biological replicates were included for each experimental group. Technical replicates were not employed, as the study focused on capturing biological variability rather than technical variation.

### Enrichment analysis and screening of differentially expressed genes

For DEGs screening, DEseq2 (v1.4.5) was utilized, with the following criteria applied: |log2foldchange| ≥ 1 and q ≤ 0.001 (adjusted p value) [44]. GO and KEGG annotations were used for functional and biological pathway classifications, and phyper function based on hypergeometric test was used for enrichment analysis. Extremely enrichment was classified as a p < 0.001, and significant enrichment as p ≤ 0.05.

### Trend analysis

Gene expression pattern analysis was used to group genes that showed similar expression patterns across several samples (at least three in a given time period). To examine the expression pattern of DEGs, the expression data of each tissue sample was clustered using the Short Time-Series Expression Miner (STEM) software (http://www.sb.cs.cmu.edu/stem/). Standardized expression data were obtained for v1/v0, v2/v0, and log2. The significant clustered profiles were shown by a p value of less than 0.05. To get more insight into the importance of the DEGs in each profile, GO and KEGG pathway enrichment studies were performed using DAVID. GO terms or pathways having a q value less than 0.05 were considered statistically significant enriched.

## Results

### Alignment analysis and data sequencing

Using the MGI-2000 platform, transcriptome libraries for the YFP sample were constructed producing 455,560,000 raw reads. 444,820,000 clean reads were recovered after low-quality, sequencing adapter-containing reads and reads with an unknown base ‘N’ content were eliminated. The sample size was over 6.71 GB, with 13 samples having base quality scores of Q20 ≥ 98 % and Q30 ≥ 95 %. When the clean read from each sample was matched to a reference genome, the average alignment rate for uniquely mapped reads was found to be 67.27 %, and for clean reads it was 89.79 % (Table 1). Given the good quality of the transcriptome sequencing, the created libraries can be used for further gene expression investigation.

**Table 1:** The Yangtze finless porpoise’s blood samples sequencing and mapping statistics.

Sample	Raw reads (M)	Clean reads (M)	Clean bases	Q20( %)	Q30( %)	Total mapped	Uniquely mapped
TT	51.99	51.63	7.61E+09	98.49	95.31	89.49	64.42
T21M17	47.46	47.11	6.97E+09	98.44	95.14	90.52	69.93
T21M12	46.62	46.12	6.83E+09	98.52	95.39	87.36	64.65
T21M09	46.39	46.09	6.8E+09	98.55	95.44	88.49	62.53
T21F19	46.37	46.04	6.82E+09	98.48	95.27	90.44	69.39
T21F18	46.00	45.63	6.76E+09	98.39	95.06	91.8	74.17
T21F12	48.74	48.37	7.16E+09	98.38	95.04	90.22	69.42
T21M06	50.16	49.78	7.36E+09	98.48	95.27	89.39	63.72
T21M03	45.78	45.40	6.74E+09	98.4	95.11	89.88	69.71
T21F04	51.47	51.12	7.55E+09	98.53	95.42	89.88	65.39
T21F02	47.84	47.50	7E+09	98.42	95.12	89.94	65.45
T21F01	45.58	45.23	6.71E+09	98.43	95.18	89.94	67.66
T21M05	50.57	50.25	7.45E+09	98.48	95.25	89.88	68.1

### DEGs analysis and functional annotation

**Fig. 1:**
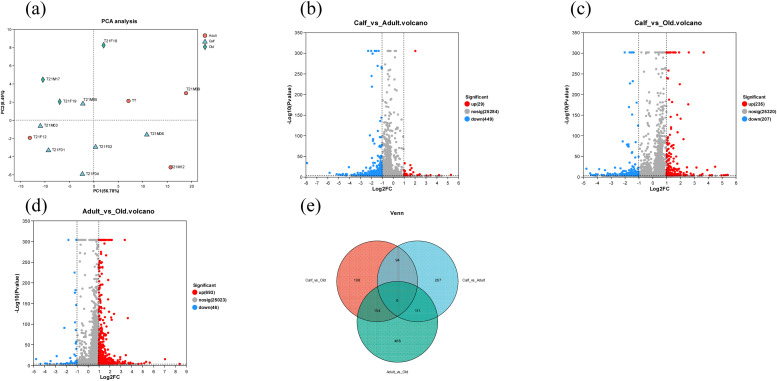
DEGs in the critically endangered YFP. (a) Gene expression among 13 samples using PCA. (b) Volcano plot between calf vs. adult, (c) volcano plot between calf vs. old, and (d) volcano plot between adult vs. old, representing each gene’s distribution of expression plotted against its log2 fold change. Genes that are differentially expressed (FDR ≤ 0.05) are represented by reads and blue dots, while non-differentially expressed genes are indicated by gray dots. (e) The distribution of common DEGs in comparison between the three groups.

**Fig. 2:**
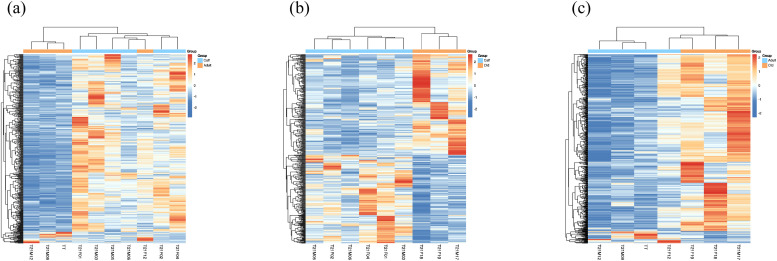
Heat map plot of up-regulated and down-regulated genes using FPKM expression value in (a) calf vs. adult group, (b) calf vs. old group, and (c) adult vs. old group respectively in the Yangtze finless porpoise.

The study identified expression genes among three age groups: calf, adult, and old YFP [69]. Principal component analysis (PCA) findings reveal that calf, adult, and old YFPs may be categorized into three groups (Fig. 1a). A total of 478 DEGs, consisting of 29 down-regulated genes and 449 up-regulated genes, were found in the calf vs. adult comparisons as shown in Table S2 and Fig. 1b 442 DEGs, comprising 235 up-regulated genes and 207 down-regulated genes, were discovered in the comparison of calf vs. old as shown in Table S3 and Fig. 1c. 739 DEGs were found when comparing the expression of 693 up-regulated genes and 46 down-regulated genes in adult vs. old YFPs as shown in Table S4 and Fig. 1d We used Venn diagram analysis (Fig. 1e) to determine the most common DEGs in the three age groups. The up-regulated and down-regulated genes were represented by hierarchal clustering analysis in the calf vs. adult, calf vs. old, and adult vs. old groups as shown in Fig. 2a–c.

The present study was carried out to investigate the physiological processes regulated by DEGs utilizing the GO and KEGG databases. The study identified 998 DEGs in the GO database, categorized into biological processes (1992), cellular components (1913), and molecular function (1241). The “molecular function” category contained the greatest number of annotated genes, all of which were involved in binding as shown in Figure S1. The KEGG database found 308 DEGs, in six functional categories i.e., genetic information processing (66), environmental information processing (465), organic systems (632), cellular processes (244), human diseases (1009), and metabolism (154). As a component of processing environmental information, signal transduction is the pathway with the greatest number of annotated genes as shown in Fig. S2.

### Gene ontology enrichment analysis of DEGs

**Fig. 3:**
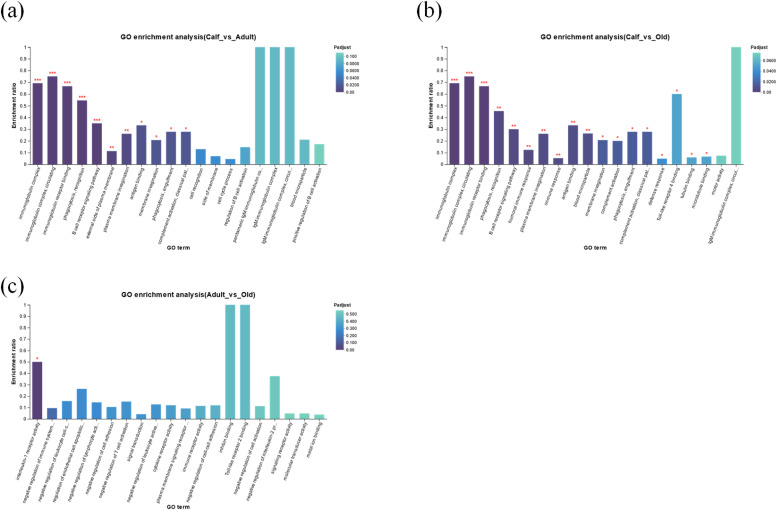
GO enrichment analysis of DEGs in the critically endangered YFP. (a) Calf vs. adult, (b) Calf vs. old, and (c) Adult vs. old. *p < 0.05, **p < 0.01, ***p < 0.001.

The study analyzed GO enrichment on DEGs to understand their biological roles. Results presented that DEGs were substantially enriched in B cell receptor signaling pathway, immunoglobulin complex, phagocytosis recognition, antigen binding, circulating immunoglobulin complex, engulfment, classical pathway, phagocytosis, complement activation, and plasma membrane invagination compared to the calf vs. adult group (Fig. 3a, Table S5). The DEGS were substantially enhanced in immunoglobulin receptor binding, circulating, immunoglobulin complex, B cell receptor signaling pathway, phagocytosis recognition, humoral immune response, plasma membrane invagination, immune response, antigen binding, phagocytosis, engulfment, defense response and other eight GO terms compared to the calf vs. old group (Fig. 3b, Table S6). The DEGS were significantly enriched in the interleukin-1 receptor activity term compared to the adult vs. old group (Fig. 3c, Table S7).

### KEGG pathway enrichment analysis of DEGs

**Fig. 4:**
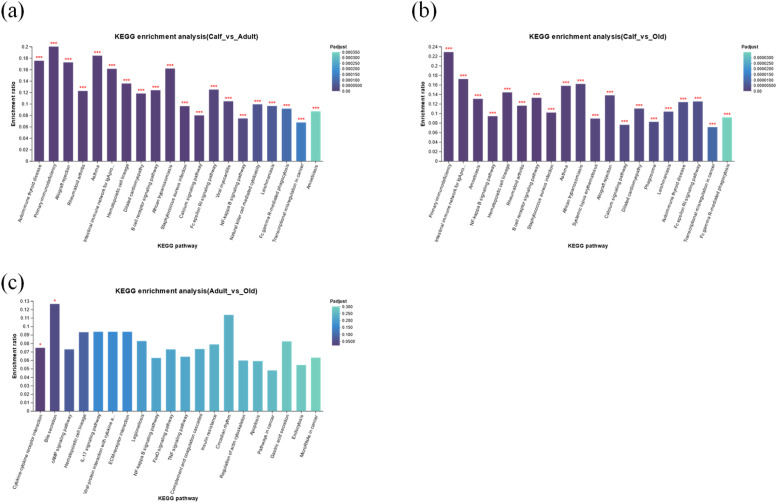
KEGG enrichment analysis of DEGs in the critically endangered YFP. (a) Calf vs. adult, (b) Calf vs. old, and (c) Adult vs. old. *p < 0.05, **p < 0.01, ***p < 0.001.

The biological significance of DEGs was further assessed through a KEGG pathway enrichment analysis. It was observed that compared to the calf vs. adult group, the DEGs were substantially enriched in primary immunodeficiency, intestinal immune network for IgA production, NF-kappa B signaling pathway, Fc epsilon RI signaling pathway, autoimmune thyroid disease, rheumatoid arthritis, B cell receptor signaling pathway, calcium signaling pathway, phospholipase D signaling pathway, natural killer cell mediated cytotoxicity, transcriptional mis regulation in cancer, Fc gamma R-mediated phagocytosis, and alpha-linolenic acid metabolism (Fig. 4a, Table S8). Compared with the calf vs. old group, the DEGS were substantially enhanced in primary immunodeficiency, NF-kappa B signaling pathway, intestinal immune network for IgA production, hematopoietic cell lineage, B cell receptor signaling pathway, cytokine-cytokine receptor interaction, Fc gamma R-mediated phagocytosis, calcium signaling pathway, inflammatory mediator regulation of TRP channels, natural killer cell mediated cytotoxicity, phospholipase D signaling pathway, complement and coagulation cascades, IL-17 signaling pathway, viral protein interaction with cytokine and cytokine receptor, TNF signaling pathway, and mineral absorption (Fig. 4b, Table S9). Compared with the adult vs. old group, the DEGS were significantly enriched in cytokine-cytokine receptor interaction and bile secretion (Fig. 4c, Table S10).

### STEM analysis in the three-age groups of YFP

**Fig. 5:**
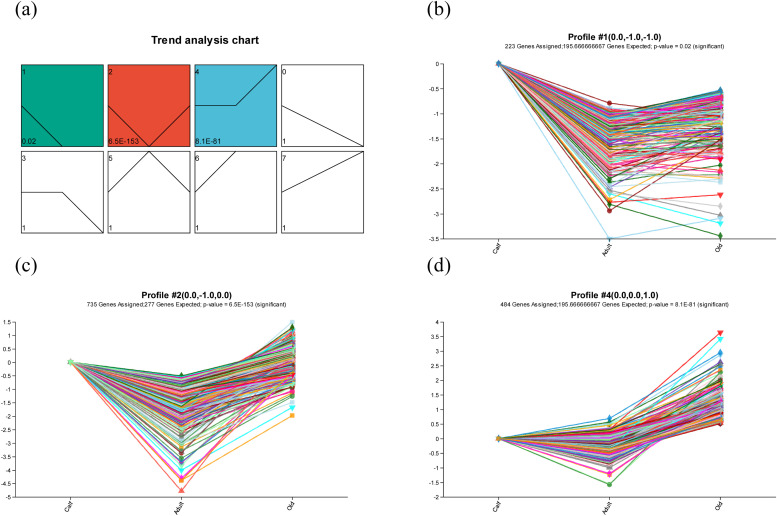
All the DEGs in the age groups of YFP were analyzed by STEM. (a) The cardinality of each cluster is shown by the number in the upper right corner, and the adjusted p value is indicated by the number in the lower left corner. All significant profiles are based on the p values of the gene counts. Expression patterns of (b) profile 1, (c) profile 2, and (d) profile 4. *p < 0.05, **p < 0.01, ***p < 0.001.

We used STEM to identify the temporal gene expression profiles and normalized the expression data of DEGs in order to show the dynamic gene expression pattern in age groups. According to the 1638 DEGs in each of the three stages, STEM categorized all DEGs into eight distinct profiles (Fig. 5a). Profile 1, 2, and 4 showed significant enrichment (p < 0.05), with profile 2 containing the highest number of DEGs.

**Fig. 6:**
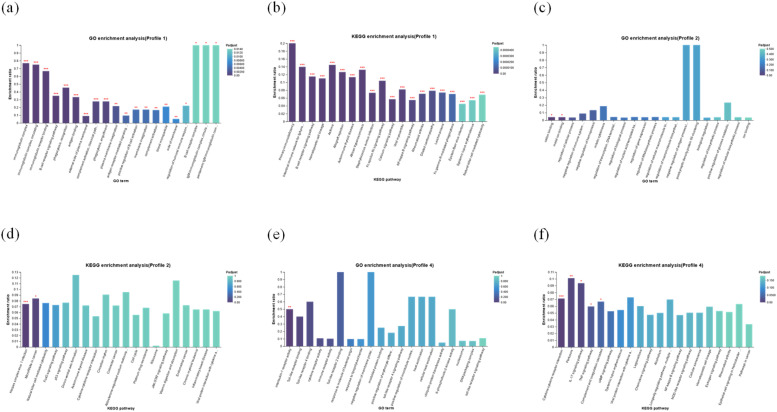
DEGs significantly enhanced the KEGG and GO pathways in each of the profiles 1, 2, and 4. (a) GO and (b) KEGG enrichment analysis in profile 1, (c) GO and (d) KEGG enrichment analysis in profile 2, (e) GO and (f) KEGG enrichment analysis in profile 4. *p < 0.05, **p < 0.01, ***p < 0.001.

The study found that genes in profile 1 were linked to 32 GO terms, including immunoglobulin complex, B cell receptor signaling pathway, classical pathway, complement activation, and IgM immunoglobulin complex, circulating and 28 KEGG pathways, including primary immunodeficiency, B cell receptor signaling pathway, intestinal immune network for IgA production, arachidonic acid metabolism, calcium signaling pathway, phospholipase D signaling pathway and 22 more (Fig. 6a, b). The study found that genes in profile 2 were linked to 2 GO terms, including cation binding and metal ion binding and 2 KEGG pathways, including MicroRNAs in cancer and Herpes simplex virus 1 infection (Fig. 6c, d). The study found that genes in profile 4 were linked to 1 GO terms, i.e., interleukin-1 receptor activity and 5 KEGG pathways, including Cytokine-cytokine receptor interaction, TNF signaling pathway, IL-17 signaling pathway, and complement and coagulation cascades (Fig. 6e, F).

## Discussion

Blood, a complex connective tissue, is used by researchers to study molecular biomarkers and evaluate animal function at any functional state, age, and environmental conditions [1,38,53]. Blood samples are easily available, allowing for extensive analyses of the blood transcriptome, which represents expression profiles linked with physiological changes in other tissues [46]. Blood samples have been utilized in previous research to investigate the mechanisms of growth and aging as well as the relationship between age and gene expression [62]. PCA results reveal that calf, adult, and old YFPs may be categorized into three groups, however, the two samples in the adult group had similar gene expression patterns, but sample T21F12 differed and resembled the calf group. While the specific process causing the unique expression pattern of T21F12 remains unknown, our data indicate that different individuals at the same age have different expression patterns. Similar differences in the adult group of pandas were found by Huang et al. [27] and the YFP by Yin et al. [69]. The KEGG pathway enriched with DEGs is predominantly associated with human diseases, suggesting age-related variations in the risk of YFP suffering from these diseases as shown in Figure S2. This finding aligns with [14], who reported that KEGG pathways are disease-related in pandas. The pathways are enriched in viral, cancer, bacterial, and immune-related subcategories. The expression of DEGs in other cancer subcategories decreases continuously with age. Recent research has shown the existence of viral [41,74] and bacterial diseases [42,43] in the YFP and ovarian and testicular cancer in old giant pandas [19]. These findings suggest that the risk of bacterial, viral, and cancer in animals changes with age. The current study indicated that the risk of viral, bacterial, and cancer in YFP increases with age, offering insights into gene expression changes and laying the groundwork for future research on molecular mechanisms underlying immune system changes with aging.

The study found that genes such as CD79B, JCHIAN, ITGA8, RYR1, KITLG, and NCKAP1 were significantly downregulated, while GPAT3 was substantially upregulated in the calf compared to the adult group as shown in Supplementary Table S2 and Fig. 1b CD79B is involved in the cell-surface of B-cell receptor (BCR) and is involved in the recognition and signal transduction of pathogens and foreign substances in B cells [8]. The study aligns with Xin et al. [65], which found downregulation of CD79B in the lung and gluteus transcriptome of yaks. This suggest that B-cell function declines in adult and old age compared to the young animals. In the calf group, the JCHAIN was also downregulated; however, its expression increase d gradually in the spleen, bone, and marrow organs that produce adaptive immune cells and may be linked to inflammation related to aging [56]. ITGA8, a gene involved in cellular processes such as activation of cell signaling pathways, cytoskeletal rearrangement, and cell adhesion [66], is downregulated in calf compared to the adult group, which may have an impact on the development and growth of calf YFP. Huang et al. [26] reported that ITGA8 is involved in the growth and development of goose muscle. RYR1, a gene involved in muscle contraction [52], was downregulated in calf compared to adults possibly affecting muscle contraction. KITLG, a tyrosine-kinase receptor ligand, is crucial for cellular development processes like neurogenesis, neuroprotection, and hematopoiesis [59,71], was downregulated in the calf group, potentially influencing cellular processes. NCKAP1 is a gene that regulates intracellular processes like apoptosis, migration, and invasion and is crucial in disease pathogenesis [68]. It may play key roles in regulating bone formation [68]. This gene might have a role in the calf YFP’s bone formation. GPAT3, a member of the AGPAT family, was upregulated in adult group and may control neuronal development [21].

The study revealed that genes like CD7, CCR7, and OASL were substantially downregulated in the calf compared to the old group, while genes like CXCL8, LY96, OSM, S100, and S100A12 were significantly upregulated as shown in Supplementary Table S3 and Fig. 1c. The gene CCR7, involved in T cell migration and memory T cell formation [7,12,30], was found to be higher and downregulated in calf compared to the old group, whose deficiency was previously associated with aging in meningeal immune cells [9]. Reduced glymphatic inflow and cognitive impairment were the results of CCR7 deficiency, which mimicked aging-associated alterations in aged YFP. Genes involved in antiviral response, such as OASL, were downregulated in calf group compared to the old and were found to be higher in adult red pandas [45]. OASL enhances innate host defense by increasing the sensitivity of RIG-I activation [76]. The genes related to humoral immune response, such as CXCL8, were also upregulated in the calf group. CXCL8, a gene coding for interleukin 8 (IL8/CXCL8), is a cardiovascular biomarker associated with obesity [2,31]. It is linked to endothelial dysfunction and atherosclerosis pathogenesis [58]. Obesity-related individuals have higher circulating IL8 levels, which positively correlate with BMI and waist circumference [31]. Lymphocyte antigen 96 (LY96) is a key component of innate immunity and is linked to neurodegenerative diseases like Alzheimer’s disease (AD) [37]. Its high expression increases oxidative stress and neuroinflammation, making it a potential target for AD treatment [15]. The increased level and upregulation of LY96 in the old group may increase the risk of AD. S100 protein are important in the etiology and inflammatory response of rheumatoid arthritis (RA) and are recognized as biomarkers of inflammation in numerous disorders [64]. The increase and upregulation of S100 genes in the old suggest that old YFP may suffer from many diseases related to arthritis. CD19 levels decrease in calf compared to the old group, leading to a significant reductio in antibody production with age [29]. Additionally, CD19 levels are reduced in captive adults compared to semi-natural adult YFPs. This suggests that age-related antibody production may decrease.

The study found that genes like IL18R1, IL18RAP, IL1R1, IL1R2, IL1RAP, and TNFSF8 were significantly increased and upregulated in the old YFP compared to the adult YFP as shown in Supplementary Table S4 and Fig. 1d IL1R1 and IL18R1 are receptors for pro-inflammatory cytokines, which induce and maintain the inflammatory cascade. IL18R1 levels may be causally associated with inflammatory bowel disease (IBD) [25]. IL18RAP is another gene upregulated in the old, playing a role in IBD, lupus, and coronary artery disease [23,54]. TNFSF8, a member of the TNF superfamily, plays a crucial role in apoptosis, cell survival, and neuroprotection [33,70]. TNSF8 was previously reported to be increased with age in humans and is associated with IBD in East Asians [10]. TNFSF8 has been linked to aging [60] and may potentially lower human lifespan [72]. Upregulation of TNSF8 in the old group may be due to aging.

The GO and KEGG enrichment analysis revealed significant changes in immune-related and signal transduction pathways such as immune response, B cell receptor signaling, immunoglobulin complex, MHC class 1 protein complex, receptor binding, phagocytosis recognition, antigen binding, complement activation, classical pathway, calcium signaling, phospholipase D signaling, hematopoietic cell lineage, intestinal immune network, and primary immunodeficiency among the three age-related groups. Additionally, we divided the expression patterns of all age-related DEGs using STEM software. STEM analysis reveals significant changes in the GO and KEGG enrichment analysis, which are mostly related to immune, metabolism, and development, and confirms the altered pathways reported in the present study by GO and KEGG enrichment analysis. The expression level of DEGs (CD19, TNFRSF13, and CD79A) enriched in the primary immune deficiency pathway was significantly decreased from calf to the adulthood and then stabilized, revealing that the innate immune response of the YFP was significantly improved from calf to adult group with development, which is in accordance with Huang et al. [27], reported gradual increase of innate immunity with age in the pandas. The adaptive immune system consists of B cell receptor signaling and humoral immune response. B cell receptor genes (B cell receptor genes CD79A, CD19, CD79B, and CR2) were significantly increased in the calf, decreased in the adult, and then increased significantly in the old, which is in line with [45] reported significant increase in the adaptive immunity with aging in pandas. While humoral immune gene (JCHAIN) was significantly increased in calf and then decreased with aging, which is also in line with Luo et al. [45] reported increase humoral genes in the calf and then decreased with aging in the pandas. The genes PTGIS, GPX7, PLA2G4F, and PLA2G12A were significantly changed in profile 1, which were increased in the calf group and significantly altered arachidonic acid metabolism, which play an important role in growth and development [57]. Increased levels of these genes may play an important role in the growth and development of the YPF. The genes IL18R1, IL18RAP, IL1R1, IL1R2, and IL1RAP were significantly upregulated in the old group and increased in profile 4, which altered the interleukin-1 receptor activity and cytokine-cytokine receptor interaction. These genes were potentially linked to IBD, lupus, and coronary artery disease [23,25,54], which may cause significant diseases in the YFP with aging, while cytokine genes play a role in the aging process [28].

The YFP is critically endangered, and this study is limited by the availability of only thirteen individuals for blood sample collection, which restricts our ability to draw a definitive conclusion about aging. In the future, we plan to increase the sample size and include more animals to strengthen our findings and better assess the impact of aging on the growth, development, and metabolism of the YFPs.

## Conclusion

The present study analyzed the transcriptomes of thirteen semi-natural YFPs, dividing them into calf, adult, and old age groups. Comparing DEGs, it was found that age stages showed significant biological differences. The study found that YFPs, which undergo significant changes in immune-related genes with age, developed immunity faster from calf to adulthood. STEM analysis was performed on all DEGs from the three groups and were involved in immune and metabolism-related pathways. The KEGG pathway, enriched with DEGs, is linked to human diseases and age-related variations in the risk of YFP suffering from these diseases. The pathways are enriched in viral, bacterial, cancer, and immune-related subcategories. The expression of DEGs in other cancer subcategories decreases continuously with age. The current study indicates that the risk of viral, bacterial, and cancer-related disease in YFP increases with age, offering insights into gene expression changes and laying the groundwork for future research on the molecular mechanisms underlying immune system changes with aging.

## Consent for publication

Not applicable.

## CRediT authorship contribution statement

**Syed Ata Ur Rahman Shah:** Writing – original draft, Visualization, Validation, Software, Methodology, Investigation, Formal analysis, Data curation, Conceptualization. **Haobo Zhang:** Writing – review & editing, Software, Methodology, Formal analysis, Data curation, Conceptualization. **Bin Tang:** Writing – review & editing, Validation, Resources, Methodology, Formal analysis, Data curation, Conceptualization. **Dekui He:** Writing – review & editing, Supervision, Resources, Project administration, Funding acquisition, Conceptualization. **Ghulam Nabi:** Writing – review & editing, Methodology. **Jinsong Zheng:** Writing – review & editing, Supervision, Software, Resources, Project administration, Methodology, Funding acquisition, Conceptualization. **Yujiang Hao:** Writing – review & editing, Supervision, Resources, Project administration, Methodology, Funding acquisition, Conceptualization.

## Declaration of competing interest

The authors declare that they have no known competing financial interests or personal relationships that could have appeared to influence the work reported in this paper.

## Data Availability

The raw data supporting the findings of this study have been deposited in the National Center for Biotechnology Information (NCBI) under the BioProject accession number PRJNA1237879.

## Appendix Supplementary materials

Image, application 1

Image, application 2
