# Supplementary material for: Comparative blood transcriptome analysis reveals changes in immunity, and transcripts related to metabolism and development in the critically endangered Yangtze finless porpoise (Neophocaena asiaeorientalis asiaeorientalis) with age
Source: Comp Immunol Rep. 2025 Oct 3;9:200255. doi: 10.1016/j.cirep.2025.200255 (PMC12547954; doi:10.1016/j.cirep.2025.200255)
Supplement: Supplementary file 1 [file mmc1.docx]

**Figure S1:** (A) GO and (B) KEGG classification of genes in the critically endangered YFP.

**Table S1:** Summary of blood samples of the Yangtze finless porpoise.

| **Sample ID** | **Animals** | **Sex** | **Sample date** | **Age** | **Group** |
| --- | --- | --- | --- | --- | --- |
| TT | TT | Male | 2021 | 13 | Adult |
| T21M17 | T21M17 | Male | 2021/4/23 | 17 | Old |
| T21M12 | T21M12 | Male | 2021/4/23 | 12 | Adult |
| T21M09 | T21M09 | Male | 2021/4/22 | 09 | Adult |
| T21F19 | T21F19 | Female | 2021/4/26 | 19 | Old |
| T21F18 | T21F18 | Female | 2021/4/26 | 28 | Old |
| T21F12 | T21F12 | Female | 2021/4/25 | 12 | Adult |
| T21M06 | T21M06 | Male | 2021/4/22 | 06 | Calf |
| T21M03 | T21M03 | Male | 2021/4/22 | 03 | Calf |
| T21F04 | T21F04 | Female | 2021/4/22 | 04 | Calf |
| T21F02 | T21F02 | Female | 2021/4/22 | 02 | Calf |
| T21F01 | T21F01 | Female | 2021/4/22 | 01 | Calf |
| T21M05 | T21M05 | Male | 2021/4/22 | 05 | Calf |
